# Tissue-Nonspecific Alkaline Phosphatase—A Gatekeeper of Physiological Conditions in Health and a Modulator of Biological Environments in Disease

**DOI:** 10.3390/biom10121648

**Published:** 2020-12-08

**Authors:** Daniel Liedtke, Christine Hofmann, Franz Jakob, Eva Klopocki, Stephanie Graser

**Affiliations:** 1 Institute for Human Genetics, Biocenter, Julius-Maximilians-University Würzburg, 97074 Würzburg, Germany; eva.klopocki@uni-wuerzburg.de; 2 Section of Pediatric Rheumatology and Osteology, University Children’s Hospital of Würzburg, 97080 Würzburg, Germany; hofmann_c5@ukw.de; 3 Bernhard-Heine-Center for Locomotion Research, Julius-Maximilians-University Würzburg, 97076 Würzburg, Germany; f-jakob.klh@uni-wuerzburg.de (F.J.); s-graser.klh@uni-wuerzburg.de (S.G.)

**Keywords:** TNAP, hypophosphatasia, HPP, zebrafish, mineralization, *ALPL*, craniosynostosis, teeth, nervous system

## Abstract

Tissue-nonspecific alkaline phosphatase (TNAP) is a ubiquitously expressed enzyme that is best known for its role during mineralization processes in bones and skeleton. The enzyme metabolizes phosphate compounds like inorganic pyrophosphate and pyridoxal-5′-phosphate to provide, among others, inorganic phosphate for the mineralization and transportable vitamin B6 molecules. Patients with inherited loss of function mutations in the *ALPL* gene and consequently altered TNAP activity are suffering from the rare metabolic disease hypophosphatasia (HPP). This systemic disease is mainly characterized by impaired bone and dental mineralization but may also be accompanied by neurological symptoms, like anxiety disorders, seizures, and depression. HPP characteristically affects all ages and shows a wide range of clinical symptoms and disease severity, which results in the classification into different clinical subtypes. This review describes the molecular function of TNAP during the mineralization of bones and teeth, further discusses the current knowledge on the enzyme’s role in the nervous system and in sensory perception. An additional focus is set on the molecular role of TNAP in health and on functional observations reported in common laboratory vertebrate disease models, like rodents and zebrafish.

## 1. Basic Information on Tissue-nonspecific Alkaline Phosphatase (TNAP/Tnap)

Alkaline phosphatases are specific enzymes present in nearly all living organisms catalyzing the dephosphorylation of pyrophosphate (PPi) and pyridoxal-5′-phosphate (PLP) in vivo, presumably as well as nucleotides like ATP and proteins like osteopontin [1,2,3].

### 1.1. Genetic Information on ALPL

Humans have four different genes encoding distinct alkaline phosphatase isoforms [4]. Three of these are considered as being expressed in a tissue-specific manner in the placenta, intestine, and germ cells (*ALPP* (NCBI GeneID: 250), *ALPI* (NCBI GeneID: 248), and *ALPG* (NCBI GeneID: 251)) and one is characterized as tissue-nonspecific (*ALPL* (NCBI GeneID: 249)). However, according to ENSEMBL and MGI databases, mice have five different genes coding for alkaline phosphatases, as the tissue-nonspecific isoform is encoded by the gene *Alpl*/*Akp2* (ENSMUSG00000028766, MGI: 87983), and additionally, *Akp3* (ENSMUSG00000036500, MGI: 87984), *Alppl2* (ENSMUSG00000026246, MGI: 108009), *Akp1* (NCBI Gene ID: 109899, MGI: 87982, not included in current mouse ENSEMBL genome annotation release), and *Alpi* (ENSMUSG00000079440, MGI: 1924018) are prevalent. According to the ENSEMBL and ZFIN databases, zebrafish have two duplicated forms of genes coding for intestinal alkaline phosphatases *alpi.1* (ENSDARG00000015273, ZDB-GENE-050327-78) and *alpi.2* (ENSDARG00000053774, ZDB-GENE-050626-142), besides *alp3* (ENSDARG00000098063, ZDB-GENE-120919-1) and the tissue-nonspecific alkaline phosphatase *alpl* (ENSDARG00000015546, ZFIN: ZDB-GENE-040420-1).

The gene that encodes the enzyme tissue-nonspecific alkaline phosphatase (TNAP for human and mouse; Tnap for zebrafish) is thus called *ALPL* in humans, *Alpl/Akp2* in mice, and *alpl* in zebrafish (NCBI HomoloGene ID: 37314), respectively. The human *ALPL* gene is located on chromosome 1 and encodes, according to the ENSEMBL database, seven different transcripts (for details, see ENSG00000162551), including predicted transcripts, which are not protein-coding and have not been analyzed in full detail. Only three human transcript variants (ENST00000374840.8, ENST00000539907.5, ENST00000540617.5) have been validated and display tissue-specific alternative splicing, e.g., in the liver and bone [5,6]. The reference transcript (ENST00000374840.8; NM_000478.6) spans a genomic region of more than 50 kb, contains overall 12 exons, including 11 protein-coding exons, has a transcript length of 2536 bp, and corresponds to the bony-type *ALPL* transcript described by Weiss et al. [6]. The murine *Alpl* gene is located on chromosome 4, encodes 8 different ENSEMBL database transcripts, while the reference transcript (ENSMUST00000030551.10; CCDS18821) contains overall 12 exons, 11 coding exons, and has a transcript length of 2522 bp. However, the zebrafish *alpl* gene is located on chromosome 11, encoding for only three transcript versions (ZFIN gene ID: ZDB-GENE-040420-1). The zebrafish ENSEMBL database reference *alpl* transcript (ENSDART00000146461.3) contains 12 exons that are all coding and has a transcript length of 1871 bp (genome assembly: GRCh38.p13, GRCm38.p6, GRCz11; summarized in [7] and [8]).

### 1.2. Biochemical Information on TNAP

**Figure 1:**
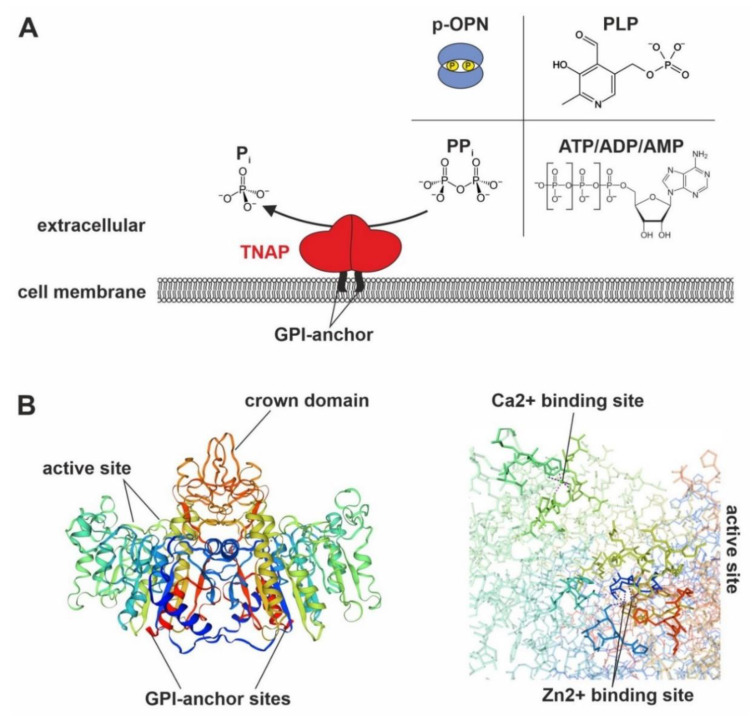
Potential enzymatic functions and structural visualization of TNAP. (**A**) Depiction of potential TNAP substrates. (**B**) Schematic depiction of a predicted homology model of TNAP’s dimerized 3D structure (https://swissmodel.expasy.org/; SWISS-MODEL: P05186 (PPBT_HUMAN); homology model according to the template: 3mk1.1.B “Refinement of placental alkaline phosphatase complexed with nitrophenyl”) and magnified view of the active site [26,27,28]. Figure 1B was modified according to SWISS-MODEL database terms of use and is licensed under a Creative Commons Attribution 4.0 (https://creativecommons.org/licenses/by-sa/4.0/). ATP/ADP/AMP: adenosine tri-/di-/monophosphate, GPI-anchor: glycosyl-phosphatidylinositol anchor, Pi: phosphate, PLP: pyridoxal-5′-phosphate, p-OPN: phosphorylated osteopontin, PPi: inorganic pyrophosphate, TNAP: tissue-nonspecific alkaline phosphatase.

TNAP is an ectoenzyme that is primarily prevalent as a dimer, anchored within the plasma membrane via a glycosyl-phosphatidylinositol (GPI)-anchor, and oriented towards the extracellular space [9]. Additionally, TNAP can be considered as a glycoprotein due to its posttranslational modifications in the form of N-glycosylations [10]. Typical substrates are inorganic pyrophosphate (PPi) and pyridoxal-5′-phosphate (PLP) as well as probably phosphorylated osteopontin (p-OPN), and adenosine tri-/di-/mono-phosphate (ATP/ADP/AMP) (see Figure 1A) [1,3]. TNAP’s structure as a GPI-anchor protein determines its recruitment to subcellular sites and extracellular vesicles, where it can influence microenvironmental concentrations of its substrates and products [1,11,12]. There is emerging evidence that the stability of the GPI-attachment determines its efficacy in the extracellular space and in the vicinity of osteoblast derived vesicles [13,14,15]. Patient mutations situated close to the GPI-anchor-encoding sequences are postulated to modulate the half-life of the enzyme on the cell surface and are, therefore, intensively investigated [16]. Several partially protein-specific phospholipases, like the phosphatidylinositol-specific phospholipase C (PI-PLC) and D (PLD), are known to cleave the connection between a corresponding protein and its GPI-anchor in the cell membrane, thus, shedding the respective proteins into the extracellular fluid compartments and the circulation [17,18,19,20,21]. In the case of TNAP, PI-PLC may play the most important role in the release of the TNAP enzyme from the cell membrane [17,21]. Linked to this release is the difficulty to provide sufficient concentration of the enzyme in the microenvironments of mineralizing tissues, which posed a challenge to manufacturing efficient recombinant enzyme preparations [1]. In the case of asfotase alfa, an artificial therapeutic TNAP enzyme, the addition of an aspartate decamer was necessary to enable the binding of the recombinant enzyme to the site of mineralization [13,22].

As depicted in Figure 1B, a TNAP dimer, which is illustrated by a structural TNAP homolog obtained from modeling PLAP, usually contains a “crown domain” mediating the interaction with the extracellular matrix (ECM), the GPI-anchor site, which is important for the membrane anchorage, and the biochemically active site, with two Zn^2+^ ions and one Mg^2+^ ion [23]. An additional Ca^2+^-binding site has been discovered in human and murine TNAP but does not seem to have direct implications on the enzyme’s catalytic activity [24,25]. However, remodeling the human mutation R206W, which is, according to the authors, located in close proximity to this area, leads to loss of enzymatic activity [15]. The dimerization interaction between two TNAP monomers, which is mediated by two disulfide bonds, can further play a central role concerning the severity of HPP symptoms due to the possibility of dominant-negative effects of an *ALPL* mutation, inherited as an autosomal dominant trait [23].

## 2. Hypophosphatasia—Diagnosis and Treatment (an Update)

Hypophosphatasia (HPP; ORPHA 436, OMIM: *171760) is a rare inherited disorder caused by loss-of-function mutations in the *ALPL* gene, encoding the phosphatase Tissue-nonspecific alkaline phosphatase (Gene Mutations Database (TNAP): http://www.sesep.uvsq.fr/03_hypo_mutations.php) [29,30,31]. It is a multisystemic disease with the involvement of different tissues and organs, including bone, muscle, kidney, lung, gastrointestinal tract, and the peripheral/central nervous system [32,33,34]. The clinical expression is highly variable, and it affects patients of all ages [31,32,33,34,35]. There are several clinical forms of the disease ranging from lethal (very severe forms in neonates and small infants with an absence of mineralization) to very mild (dental abnormalities, mild musculoskeletal problems in children or adults) [36,37,38]. The main clinical signs and symptoms are related to defective bone and tooth mineralization (rickets-like deformities, osteomalacia, fractures, premature deciduous tooth loss with intact roots, and short stature), but in severe forms, other systemic manifestations (seizures, respiratory and kidney problems, chronic pain, muscle weakness, and craniosynostosis, etc.) may be present [32,34,39,40,41].

Diagnosis is mainly based on characteristic clinical signs and symptoms in combination with consistently low age- and sex-adjusted serum alkaline phosphatase (AP) activity [33,34,41]. Additionally, elevated concentrations of plasma PLP or urine phosphoethanolamine (PEA) may be helpful to support the diagnosis as well as genetic testing for *ALPL* mutations as a confirmatory tool [31,42]. Further assessment by conventional X-rays and other imaging techniques are relevant tools for the diagnosis and follow-up [33,41,43].

Up to now, no curative treatment of HPP is available. Therefore, symptomatic treatment is very important and often requires a multidisciplinary team depending on the disease severity. Over the last decades, several supportive treatment regimens have been described for symptomatic treatment in particular with regard to pain, seizures, and other metabolic phenomena to improve the health-related quality of life (HRQoL) of patients with HPP of all ages [22,33,44,45]. Asfotase alfa (Alexion Pharmaceuticals Inc., Boston, MA, USA) is a human, recombinant bone-targeted enzyme replacement therapy that is approved for the treatment of patients with pediatric-onset HPP (to treat bone manifestations of the disease) [46,47]. The efficacy and safety of asfotase alfa have been assessed in non-comparative prospective, open-label, phase 2, multinational clinical trials in infants, adolescents, and adults with perinatal, infantile, or childhood forms of HPP [46,47,48,49,50,51]. The treatment leads to skeletal, respiratory as well as functional improvement and marks a milestone in the therapy of severe forms of HPP [22,52]. Compared to age-matched historical controls, patients with life-threatening HPP treated with asfotase alfa had substantially improved bone mineralization, survival, and ventilation-free survival [48,49,50,51]. In childhood HPP, treatment with asfotase alfa improved growth, gross motor function, strength, agility, and decreased pain [51]. A 5-year study on adults and adolescents with pediatric-onset HPP concluded that asfotase alfa is associated with enhancement of circulating TNAP substrate levels and improved functional abilities [53]. Asfotase alfa was generally well-tolerated, with most adverse events being mild-to-moderate [48,49,50,51,53]. The most frequently observed adverse reactions were injection-site reactions (in approximately 73%), and clinical trials revealed the development of anti-drug antibodies after treatment with asfotase alfa (in more than 80% of the subjects) [48,49,50,51,53]. Up to now, this has not been shown to have a discernible effect on clinical efficacy [46,47]. Very recently, recommendations for monitoring guidance for HPP patients treated with asfotase alfa have been published, based on the consensus of an international expert panel of physicians [45]. Those recommendations include laboratory, efficacy, and safety assessments in a coordinated, multidisciplinary, team-based, and patient-focused approach.

## 3. The Molecular Role of TNAP in Bone Mineralization

**Figure 2:**
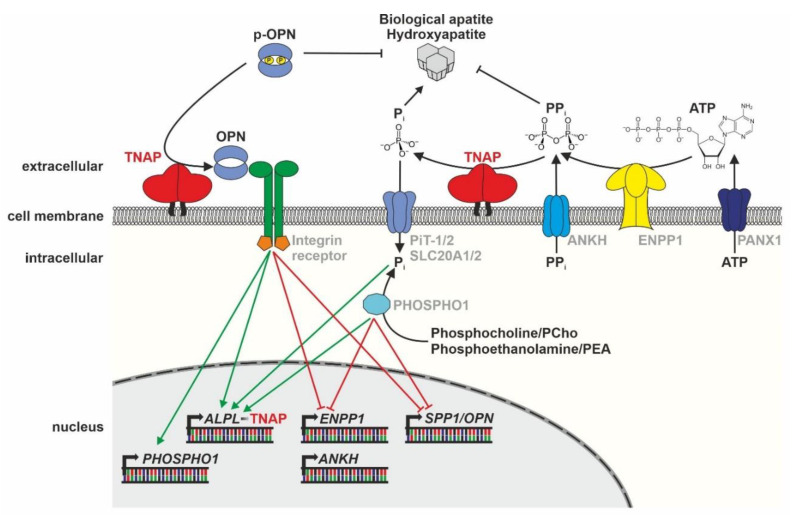
Schematic model of TNAP function within mineralizing cells (Modified according to Yadav et al. 2014 [67]). ATP: adenosine triphosphate, ANKH: progressive ankylosis protein homolog, ENPP1: ectonucleotide pyrophosphatase/ phosphodiesterase family member 1, SPP1/OPN: secreted phosphoprotein 1/osteopontin, Phospho1: phosphoethanolamine/phosphocholine phosphatase, Pi: Phosphate, PiT-1/2: sodium-dependent phosphate transporters 1 and 2, p-OPN: phosphorylated osteopontin, PPi: inorganic pyrophosphate, TNAP: tissue-nonspecific alkaline phosphatase.

As described in detail in the preceding chapter, the rare disease HPP often goes along with severe mineralization defects leading to rickets in children and osteomalacia in adults [36,37,38]. Due to the impaired mineralization of their bones, patients often suffer from an increased risk of developing fatigue fractures during their life [1]. Additionally, short stature and waddling gait are typical symptoms of HPP patients. Despite the actually anti-mineralization environment due to reduced TNAP activity, craniosynostosis, premature closure of suture vaults of the skull can occur in severely affected children [1]. These symptoms are the results of either reduced molecular functions or impaired transcriptional regulation (Figure 2).

### 3.1. Cellular Function and Regulation

The TNAP enzyme is well-known to reduce the anti-mineralization effects of PPi, to produce monophosphate (Pi) for the propagation of hydroxyapatite (HA), and to interfere with biological apatite crystallization [54,55]. Additionally, TNAP is capable of catabolizing ATP stepwise to adenosine, which is integrating the enzyme into the purinergic signaling system [1].

Regulation of mineralization (see Figure 2) is an overall quite complex process, which involves several enzymes, such as ENPP1, PHOSPHO1, ANKH, and PANX1, which control the availability of TNAP substrates and its products in the microenvironment [11,56]. Here, the prevalent concentration of the mineralization inhibitor PPi in the microenvironment is crucial, which is influenced by TNAP dephosphorylating PPi to Pi and by PPi transport via the ANKH channel to the extracellular space [11]. Furthermore, the membrane channels PANX1/3 are linked to the mineralization process [57], as they enable the translocation of ATP from the cellular to the extracellular space [56]. The phosphatase PHOSPHO1 is, among others, localized inside matrix vesicles, provides Pi for HA crystallization, and has a nonredundant function to TNAP, demonstrated by a double knockout of both genes resulting in a complete lack of mineralization in mouse models [54,58]. Controversially discussed regulators of the intracellular Pi concentrations are sodium/phosphate co-transporters, like Pit1/2 (SLC20A1/2), which have been reported to affect bone mineralization [59]. However, recent mouse knockout studies imply an additional transport-independent function of Pit1 in endoplasmatic reticulum (ER) homeostasis and chondrocyte survival [60].

The central step of bone mineralization is the budding of extracellular vesicles carrying TNAP and PHOSPHO1 from the plasma membrane of osteoblasts during the delivery process of HA crystals into the extracellular matrix [54]. Interestingly, the anchorage of TNAP to a cellular membrane, e.g., the membrane of matrix vesicles (MVs), may be able to influence the substrate specificity of the enzyme [17]. Dephosphorylation of p-OPN, a member of the integrin-binding ligand N-linked glycoprotein (SIBLING) family, is catalyzed by TNAP [3]. Secreted OPN is bound by integrins [61,62], enabling intracellular signaling changes and directing cellular behavior, like migration [63]. Intracellular signaling changes and subsequent gene expression regulation are crucial to control the occurrence of key enzymes and channels at cellular membranes and to regulate TNAP’s biochemical functionality [64]. Recent studies in *Alpl*^−/−^ mice have shown that the regulation of the expression of master osteoblast transcription factors Runx2 and Sp7 and thereby, osteoprogenitor differentiation is dependent on TNAP function [64]. Subsequent expression of enzymes, like ENPP1 [65], is directly depending on these lineage-specific transcription factors and their regulation network [66]. The following Figure 2 summarizes the main enzymes and gene regulation pathways that are involved in HA crystallization in the extracellular space of mineralizing cells.

### 3.2. Animal Models of HPP

Two independently established *Akp2* knockout (k.o.) mice (*Alpl*^tm1Sor^/*Tnap^−^* (MGI ID: 1857124) and *Alpl*^tm1Jlm^/*Akp2*^−^ (MGI ID: 2183411)) can be considered as murine models for the infantile subtype of HPP [68,69,70]. Compared to the wildtype, their TNAP activity is halved in heterozygous mice and nearly reduced to zero in homozygous k.o. mice [70]. At approximately postnatal day 10, characteristic bone abnormalities appear, which include rickets, increased number of fractures, osteopenia, and increased PPi and PEA levels in the urine as well as PLP in the plasma, in line with the situation in human patients [70]. The animals display clear pathological signs in bone histology, like a smaller hypertrophic zone in the tibial growth plate due to a developmental arrest of chondrogenesis at the age of 18 days [70]. Additional data from Bessueille et al. confirm the disturbance in chondrocyte maturation in the metaphysis of heterozygous *Akp2^+^*^/−^ mice [71]. Furthermore, *Akp2*^−/−^ mice with severe bone phenotype may potentially also develop craniosynostosis and display aberrant development of their craniofacial shape [72]. Apart from these infantile HPP mouse models, one adult HPP mouse model has been described (*Alpl*^tm2.1Jlm^/*Alpl*^A116T^), displaying a dominant-negative effect caused by the prevalent *Alpl* mutation [73]. In addition to the well-studied *Alpl* k.o. mouse models, a large number of either chemically induced ENU (N-ethyl-N-nitrosourea) or gene trap mouse mutants are described, partly showing mutation dependent variable bone and skeletal defects [74,75,76].

Zebrafish have become a popular vertebrate model for the examination of bone developmental processes and bone diseases [77,78]. Although, endochondral ossification is not as frequent as in mammals and additionally, intramembranous and perichondral ossification is present in zebrafish [79]. Investigation of TNAP function in zebrafish is still fragmented but may open up the possibility to investigate more basic developmental aspects due to its biological features, like extracorporeal fertilization, optical transparency, and numerous transgenic fluorescent reporter lines [77,80]. Genetic analyses of *alpl* gene evolution in vertebrates and their expression in zebrafish clarified that non-mammalian species possess a number of additional *alpl* genes due to genome duplication events [8]. *alpl* expression has been reported in zebrafish as a readout of osteogenic defects, e.g., in *Sp7/osterix* mutants and after ferric iron ammonium citrate treatment, although studies exclusively focusing on Tnap function in zebrafish bone development are still rare [81,82]. An incubation experiment in zebrafish using the potent chemical inhibitors levamisole and MLS-0038949 that specifically inhibit the Tnap enzyme resulted in impaired craniofacial development and bone mineralization delay or even abolishment, depending on the respective inhibitor concentration that was used for the treatment of the zebrafish larvae [7]. Future experiments must establish more precise in vivo zebrafish models that are adapted to investigate Tnap function, either by tissue-specific knockout or by introducing disease-causing mutations. These will not only be useful for functional investigations in skeletal tissues but also enable investigation of TNAP/Tnap´s role in regeneration, behavior, and evolution.

### 3.3. Combinatory Effects of TNAP in Murine Animal Models

Several mouse models reveal that PPi inhibits ectopic calcification, even if extracellular Pi is increased (here, the ratio of PPi and Pi is important), as PPi prevents Pi from forming crystals with calcium [83,84]. TNAP alone is not sufficient to initiate mineralization of the ECM, especially in the presence of high concentrations of PPi and phosphorylated osteopontin [11,85,86,87,88]. Interestingly, a simultaneous k.o. of OPN partially rescues the mineralization defects in HPP mouse models but fails to normalize the elevated PPi levels [85]. In addition, the simultaneous presence of collagen type I is important for mineralization, which is the case in bones and teeth [87]. The overexpression of TNAP promotes bone mineralization due to dephosphorylation of p-OPN and degradation of PPi [3]. However, the aberrant metabolism of vitamin B6 (PLP/pyridoxal (PL)) does not markedly contribute to the visible defects in bones [89]. Experiments applying the recombinant enzyme asfotase alfa to several mouse models show that the recombinant enzyme is not only able to bind to bones and teeth, thanks to its bone-targeted deca-aspartate sequence, but also to ectopic calcification spots, i.e., in blood vessels [90]. Another relevant study that analyzed k.o. mice revealed that an additional deletion of *Enpp1* normalizes the hypomineralization status that is related to increased PPi levels in *Akp^−/−^* mice [11,67], whereas *Akp2* k.o. and simultaneous point mutation in the *Ank* gene (*ank/ank* mice) only improves the phenotype to a certain extent [11]. Generally, the mineralization deficiency is more intense in *Enpp1^−/−^* mice compared to *ank/ank* mice and only NPP1 is located in the MVs [11]. However, levels of p-OPN, an inhibitor of mineralization [91,92], are increased in *Akp2^−/−^* mice, decreased in *ank/ank* mice, but at normal levels in double k.o. lines [11]. Despite the similar phenotypes of *Phospho1^−^*^/*−*^ and *Akp2^−/−^* mice due to increased levels of p-OPN and PPi, both enzymes act in a nonredundant manner (Phospho1: initiation of HA crystallization in the MVs, TNAP: the spread of HA crystallization in the ECM) [67,93]. Therefore, a *Phospho1* and *Akp2* double k.o. leads to early postnatal death and a complete lack of mineralization [58]. On the contrary, *Spp1/Opn* k.o. can largely compensate for the mineralization deficits in the vertebrae and long bones of *Phospho1^−^*^/*−*^ mice [67].

The discussion of further effects of TNAP on other diseases (as follows) may give additional input concerning the diverse molecular roles of the enzyme. For example, a recent study with *Abcc6^−/−^/Akp2^+/−^* double mutant mice suggests that decreased activity of TNAP improves the symptoms of the mineralization disease pseudoxanthoma elasticum (PXE; OMIM: # 264800) [94]. The molecular basis of PXE is a lacking release of ATP via the ATP-binding cassette subfamily C member 6 (ABCC6) in the liver, consequently leading to a decreased concentration of PPi [94]. Additionally, TNAP expression is increased in pancreatitis, supporting the notion that the influence of TNAP on inflammatory processes is not only restricted to the environment of bone [95]. Because of its ability to degrade proinflammatorily active ATP molecules, TNAP should be able to act as an anti-inflammatory enzyme; nevertheless, this effect does not seem to be of high relevance in osteoblasts, but indeed in neutrophils [71]. It is noteworthy in this context that TNAP also acts as a nucleotidase in chondrocytes but does not lead to autocrine anti-inflammatory effects in those cells [71].

## 4. The Role of TNAP in Teeth

### 4.1. TNAP Function in Human Dentition

Besides its function in bone establishment, the TNAP enzyme is also playing a central role in the mineralization process of teeth [96,97,98]. Consequently, HPP patients often develop a pathologic phenotype concerning their orodental health as a consequence of their decreased TNAP activity [97,99,100]. Goseki-Sone et al. confirmed the presence of the bone-type of TNAP in the pulp, the periodontal ligament (PDL), and the dental sac of human samples [101]. Generally, TNAP contributes to the establishment and maintenance of the orodental system in diverse manners, which is, above all, becoming obvious in the symptoms of HPP patients [100,102,103]. They display premature loss of deciduous teeth without prior root resorption, reduced mineralization of craniofacial bones, and potentially deteriorated dentin as well as enamel production [99,104]. Decreased surface of the craniofacial bones leads to diminished anchorage options for the tooth roots, which further deteriorates the anchorage situation. Additionally, periodontitis is another issue that may occur in the context of HPP [105]. Interestingly, the clinical HPP subtype that is called Odonto-HPP is negatively influencing the orodental health of the patients, but not their bone mineralization status [1].

Our own group recently discovered that in vitro chemical inhibition of TNAP in primary human stem cells from the dental pulp (DPSCs) and the PDL (PDLSCs) leads to decreased expression of core genes of osteogenesis and mineralization, decreased expression of genes related to production/turnover of the ECM, and to an increased expression of genes related to inflammation [103]. In detail, RNAseq analysis comparing PDLSCs during osteogenic differentiation with and without the TNAP inhibitor levamisole revealed increased expression of *P2X7*, *LPL*, and *CNTNAP2*, whereas *DMP1*, *WNT2*, *WNT7B*, *GABBR2*, *ELN*, and *ENPP1* were decreased due to TNAP inhibition [103]. A study conducted by Tomlinson et al. further confirmed TNAP expression in subsets of DPSCs [96]. The authors describe that those cells are simultaneously expressing typical MSC markers, like CD73 and CD90, but their expression does not depend on a prevalent TNAP expression [96]. Furthermore, the study reveals that the TNAP expression is above all present on proliferating cells and changing with respect to cell cycle status, cell density (high density leading to increased TNAP expression), and cultivation time of the cells [96]. Generally, the PPi/Pi ratio has a significant influence on the processes of cementogenesis as studies have shown that low PPi levels promote the growth of acellular cementum, whereas a high prevalence of PPi decreases its formation [106]. Therefore, mutations in the gene encoding ENPP1 are leading to low PPi levels and an increase of the cervical cementum [107]. It can additionally be assumed that disturbances within the mineralization process may also lead to pathological circumstances concerning the movement of the teeth and their exfoliation [107].

### 4.2. Vertebrate TNAP Dentition Models

On the one hand, the lack of TNAP activity in *Akp2^−/−^* mice prevents the establishment of acellular cementum. On the other hand, it promotes the setup of cellular cementum [108]. It was suggested that the early expression of TNAP leads to the initial formation of acellular cementum due to low PPi levels, whereas the late expression of ENPP1 inhibits the apposition of acellular cementum [108]. Same as in bone, MVs are also playing a central role in dentin mineralization, with TNAP being dispensable for initiation of mineralization but central for the progression of the whole process [109,110]. Treatment of the murine HPP model with recombinant TNAP enzyme leads to the prevention of HPP-associated defects in the enamel [111]. Foster et al. analyzed the defects in dentin mineralization in *Akp2^−/−^* mice during tooth development and revealed a delay in the root development of molars and incisors to diverse extents due to deviant expressions of marker genes like *Bglap/Osteocalcin* and *Dspp* (dentin sialoprotein) [98]. Overall, TNAP plays a role in the differentiation of odontoblasts, secretion of dentin matrix, and mineralization [98]. An analysis of the spatiotemporal expression of *Tnap* in rat teeth verified a high-level expression in two phases of the developmental process: during the initial mineralization phase and again in the terminal growth phase of large enamel crystals in ameloblasts, which is indeed an interesting difference compared to mineralization processes of bone matrix [112]. Additionally, the authors show that TNAP is located in the extracellular space of dentin as well as enamel, and remarkably, the distribution of the enzyme is organized in a gradient instead of the diffuse manner that can be seen in the bone ECM [112]. Those differences may influence the susceptibility of dental tissue and bones, respectively, to TNAP malfunction, as speculated by Hotton et al. [112]. *Tnap* and *Phospho1* are coordinately expressed during odontogenesis, and a simultaneous decrease of their expression leads to aberrant development of dentin [113]. Interestingly, the number of MVs is severely reduced in the mantle dentin of *Phospho1*^−/−^/*Akp2*^+/−^ mice [113]. On the contrary, it was increased in bones of a stillborn *Phospho1*^−/−^/*Akp2*^−/−^ embryo as discussed by McKee et al. [58]. Moreover, the consequences of *Phospho1* and *Akp2* ablation are clearly different between incisors and molar crown dentin, which implies an alternative developmental mechanism [113].

The prevalence of PPi is severely affecting cementogenesis, with low levels promoting the growth of acellular cementum (like in *Ank* k.o. mice) and high levels reduce the acellular cementum (like in *Akp2* k.o. mice) [106]. In addition, changes in *Enpp1* expression and consequently in the PPi concentration influence the growth of cementum [106,107], whereas a double k.o. of *Ank* and *Enpp1* neutralizes this phenomenon [106]. Taken together, the acellular cementum obviously reacts more intensely to changes in the PPi environment than the cellular one [106]. Furthermore, reduced PPi levels lead to changes in the expression of ECM proteins in the periodontium [106], which may be an explanation for the expression changes of elastin we observed in human PDLSCs treated with the TNAP inhibitor levamisole during osteogenic differentiation [103]. However, a double k.o. of *Ank* and *Akp2* improves the mineralization of alveolar bones (relative to *Akp2* k.o. alone) [106].

In contrast to the mammalian models, zebrafish have different pharyngeal dentition, and the formation of the teeth follows very strict and repeatable developmental patterns. During the development of zebrafish, the teeth are arranged in transverse rows (anterior to posterior) as well as from dorsal to ventral within a row [114]. Teeth are usually formed by the orthodentine that is located close to the pulp cavity and are covered with an enameloid cap [115]. Zebrafish are constantly replacing their teeth, and the starter tooth (named 4V1) is stimulating and probably initiating the development of the following ones in the pharynx [116]. Adult Zebrafish dentition includes three rows with five ventral teeth, four mediodorsal teeth, and two dorsal teeth on each side [117]. The first tooth is visible at approximately 2 days post fertilization (dpf) and the last one only four weeks later [117]. Interestingly, dentition develops in a very symmetric manner on both sides of the pharynx until 10 dpf [114]. The role of Tnap for the dental development of zebrafish is to date unclear, and further research needs to be performed on its possible contribution.

## 5. TNAP and Its Role in Pathologies Like Craniosynostosis and Atherosclerosis

Basically, TNAP is in charge as a gatekeeper for both the propagation of apatite crystallization and consequently the mineralization of bones, dentin, and enamel [54,118] when it is highly active. However, it also controls local PPi concentrations when low TNAP activity leads to the accumulation of the substrate PPi as a very strong inhibitor of mineralization [119]. Hence, identical mechanisms can support physiological effects for the mineralization of bones, dentin and enamel, but also pathologic effects if mineralization occurs in tissues that actually should not mineralize under normal conditions, such as the media and the atherosclerotic plaques of atherosclerotic vessels [120,121]. Paradoxical premature closure of cranial sutures is an issue that is even more complex and not yet completely understood, as discussed below.

### 5.1. Craniosynostosis

Premature closure of the cranial sutures in severely affected HPP patients is a pathologic mechanism that could be associated with disturbed TNAP activity [1]. The disease process, which is called craniosynostosis, can result in skull deformation, neurological damage, and often requires early neurosurgery in childhood [40]. It is still unclear how reduced TNAP activity leads to premature mineralization in the cranial sutures, in contrast to the decreased mineralization status observed in the rest of the body. However, the present view envisions craniosynostosis as a growth disorder rather than a mere mineralization problem [122]. In this context, it is important to take into account that the skull is composed of bones that are derived from different embryonic tissues, neural crest-derived, and paraxial mesenchyme derived skeletal precursors [123]. Moreover, the suture comprises several specific stem cell populations that are spatially organized over time and differentially contribute to the suture closure process [124,125]. Neural crest-derived cells are reported to be more proliferative and osteogenic within activated Wnt and FGF signaling domains, and both types also mutually influence each other [126]. They were shown to more readily produce mineralization nodules in comparison with mesenchyme-derived cells, possibly indicating that they can take over the lead in the suture milieu in case of altered osteogenic differentiation, thereby supporting craniosynostosis [127]. We know from inherited syndromal craniosynostoses that components of several different signaling pathways could be involved in the pathomechanism of craniosynostosis [128]. Hence, there is future research warranted to fully dissect the molecular components and pathways involved in this condition, including the role of TNAP and tissue mineralization.

Decreased TNAP expression is also leading to aberrant development of the skull in *Akp2*^−/−^ mice, analogously to the situation in human HPP patients [129]. An additional study conducted by Liu et al. revealed that calvarial cells of *Akp2*^−/−^ mice displayed decreased proliferation rates as well as increased expression rates of several genes, including *Runx2* and *Osteocalcin* under standard culture conditions, but decreased expression levels during osteogenic differentiation compared to wildtype cells [130]. Interestingly, only the proliferation defects can be rescued by administration of Pi, emphasizing that additional pathways are leading to the changes in the TNAP-deficient calvarial cells [130]. Moreover, if the *Akp2*^−/−^ mice were treated with the replacement enzyme shortly after they were born, deficits in craniofacial mineralization improved, and the occurrence of craniosynostosis could be prevented [130]. Application of TNAP using a viral delivery system was able to improve the prenatal craniosynostosis phenotype caused by an activating *FGFR2* mutation in one out of two murine Crouzon syndrome models tested [131].

Zebrafish skulls are anatomically quite different from mammalian and higher vertebrates; nevertheless, investigation of general morphogenesis of the cranial sutures and craniosynostosis is possible [132]. The zebrafish has been established as a suitable model for analyzing different disease-causing genetic alterations identified in craniosynostosis patients, like mutations in the transcription factors TCF12 or TWIST1 [133], which are known activators of Runx2 in mice [134]. Similar to humans and rodents [133], ablation of Tcf12 function results in premature closure of the coronal suture due to an imbalance in progenitor cell maintenance and differentiation in zebrafish [135]. In vivo visualization of *tcf12* expression in zebrafish further implies that expressional timing and localization on suture growth zones are conserved and that the expression of *tcf12* is regulated by evolutionary conserved genetic enhancer elements [136]. Besides the investigation of different Saethre–Chotzen syndrome mutations, zebrafish have been successfully used to investigate novel mechanisms causing craniosynostosis [137]. One example is the deregulation of retinoic acid signals by chemical inhibition or by the mutation in the *CYP26B1* gene, which results in severe limb and skeletal defects and in craniosynostosis in zebrafish [137]. The function of CYP26B1, i.e., inactivation of all-trans-retinoic acid and thereby potential regulation of concentration gradients [138], is conserved in vertebrates and is essential for osteoblast-osteocyte transition, subsequently resulting in the observed prominent malformations of extremities within in knockout mice [139]. Detailed genetic investigation of *alpl* function in zebrafish skull development and in suture formation are still missing, but the application of diverse Tnap inhibitors implies abnormal craniofacial development in zebrafish embryos [7].

### 5.2. Vascular Calcification

The pathogenesis of atherosclerosis in humans is a multifaceted disease with several factors showing a strong impact on its initiation and progression [140]. Local inflammation, recruitment of cells of the innate immune system, initiation of osteogenic differentiation pathways in smooth muscle cells of the arterial wall, and inflammatory activation of the endothelia and others are important contributors that all together support calcification in the vessel wall and in atherosclerotic plaques [140]. As the local availability of PPi is stated to be the major contributor to calcification, TNAP must be considered as a local gatekeeper [140]. HPP patients should be protected as enhanced TNAP activity through inflammation and local dystopic osteogenic activity or enzyme replacement in HPP will trigger calcification [140]. The clinical impact of calcification on cardiovascular endpoints can paradigmatically be demonstrated in coronary artery disease [141]. Although the morphology and functional impact of atherosclerotic plaques (occlusive vs. non-occlusive) must be taken into account, the mere calcium scoring of coronary arteries in high-resolution computed tomography has become an important risk factor that influences guidelines for patient management, as for example, the indication for statin treatment [141,142]. Vascular calcification is promoted by different mechanisms, including an abnormal osteogenic differentiation of vascular smooth muscle cells, the death of vascular smooth muscle cells, increased Pi levels, e.g., due to increased TNAP and Phospho1 activity or in chronic kidney disease, and chronic inflammation [120,143].

Interestingly, TNAP expression is increased and consequently promoting the propagation of ectopic calcification in blood vessels by decreasing the PPi concentration and providing Pi in a CD73 deficiency situation [144]. A recent publication describes the establishment of mice expressing TNAP in VSMSCs or endothelial cells, respectively, by crossbreeding male homozygous Tagln-Cre or heterozygous Tie2-Cre mice with female homozygous Hprt*^ALPL^* knock-in mice [90]. Those mice, as well as WT mice, were treated with a single dose of fluorescence-labeled asfotase alfa, and imaging showed that this compound bound to both skeletal and dental structures, but also to sites of ectopic calcification in the modified mice [90]. Furthermore, the development of ectopic calcification can be induced by overexpression of TNAP in endothelial cells and leads to an increase of the expression of typical osteogenic genes in a mouse model [145]. Additionally, typical consequences of arterial calcification, like increased blood pressure, occur in these mice after 23 weeks [145]. In contrast, inhibition of TNAP in a CKD mouse and a rat arteriosclerosis model ameliorates arterial calcification and improves survival [46,47].

## 6. TNAP beyond Mineralization—TNAP and Its Molecular Role in General Brain Function, Pathology, Behavior, and Pain

Similar to the situation in bone, TNAP expression is generally at the highest level during developmental processes of the nervous system and is, among others, involved in the establishment of synapses and in the maintenance of their functionality on a molecular level [146]. Severe neurological symptoms, i.e., fatal encephalopathy, have been reported for a patient suffering from perinatal HPP with a complete lack of TNAP activity as two compound heterozygous mutations were detected in the *ALPL* gene, thereby highlighting the role of the enzyme during the neurological development in humans [147] (see also chapter “Neurological symptoms of HPP”).

### 6.1. The Role of TNAP in the Development of the Nervous System

**Figure 3:**
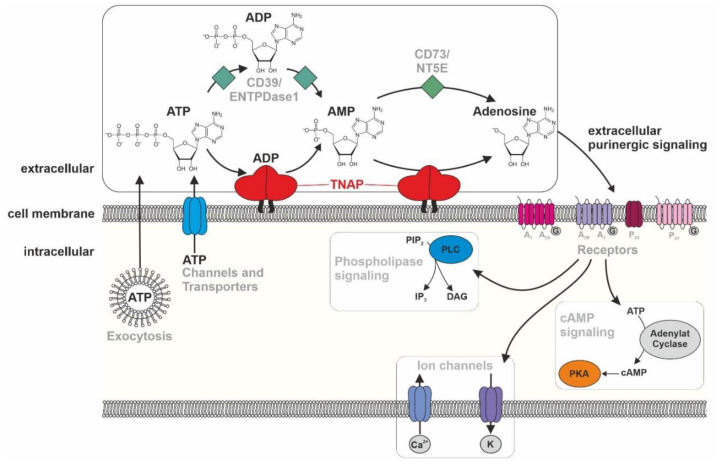
The role of TNAP within the purinergic signaling. **ATP/ADP/AMP**: adenosine tri-/die-/monophosphate, cAMP: cyclic adenosine monophosphate, DAG: diacylglycerol, CD39/ENTPDase1: ectonucleoside triphosphate diphosphohydrolase 1, IP_3_: Inositol trisphosphate, CD73/NT5E: ecto-5′-nucleotidase, PIP_2_: phosphatidylinositol 4,5-bisphosphate, PKA: phosphokinase A, PLC: phospholipase C, TNAP: tissue-nonspecific alkaline phosphatase.

TNAP is discussed to play a role in the purinergic signaling by dephosphorylation of ATP to adenosine [1,2,148], which is schematically depicted in Figure 3. ATP reaches the extracellular space either via exocytosis or via channels and transporters [148]. Subsequently, the membrane-bound ectoenzyme TNAP degrades it to adenosine, with the intermediates ADP and AMP, and consequently influences purinergic signaling pathways by either providing or decreasing ligands for purinergic receptors (P2 nucleotide receptors and P1 adenosine receptors) [148]. Additionally, the enzyme CD39 (ENDPTase1) is able to degrade ATP to AMP and CD73 (NT5E) AMP to adenosine and therefore having an impact on purinergic signaling [149,150,151,152,153]. However, the physiological impact of TNAP vs. CD39/CD73 in adenosinergic signaling may be cell type-specific and needs further clarification. The activation of diverse purinergic receptors, which are either G-protein-coupled receptors or ion channels, influences diverse processes, like cAMP signaling, PLC signaling, and the activation of K^+^ and Ca^2+^ ion channels within the cellular membrane [148].

A number of different mouse models have been reported, and the role of TNAP in murine brain development is extensively discussed in Zimmerman and Langer. Briefly, *Alpl* expression can already be detected in the blastocyst stage and constantly changes with respect to the developmental stage [154]. TNAP activity is considerably increased during the early stages of brain vesicle development at E9, followed by a decrease between E11 to E14, which is indicating a function during early phases of the development and a decrease in higher developmental stages of the murine embryo [154]. Interestingly, *Alpl* was expressed in all telencephalic ventricle cells at E14. However, later on, the expression was only detected in ventricular and subventricular zones (SVZ) [155]. In the lateral ventricles of adult mouse brains, Langer et al. detected the enzyme in the SVZ and in the rostral migratory stream [155]. More than half of the TNAP-positive cells were also positive for the microtubule-binding protein doublecortin (DCX) in the adult mouse brain, which is indicating that the majority of *Alpl* expressing cells may be immature neurons as *Dcx* is, among others, expressed in migrating cells (and in the SVZ of adult brains) [155,156]. Additionally, TNAP promotes the proliferation and differentiation of neuronal stem cells that were extracted from the subependymal layer of the lateral vesicles of 8- to 12-week old mice [157]. Narisawa et al. describe the changes of *Alpl*’s spatial expression and its intensity during murine neuronal development in detail, including the observation that the enzyme is indeed localized to neuronal cells [158]. Investigation in rats further imply that 17β-Estradiol promotes the outgrowth of dendritic spines in the hippocampus and its systemic application leads to downregulation of NTPDase1 and NTPDase2 as well as to upregulation of TNAP activity, which is indicating an interaction of the purinergic and estradiol signaling pathways during rearrangements of dendritic spines [159,160]. Analyses of *alpl* expression in neuronal tissues during zebrafish embryogenesis via in situ hybridization (ISH) are described in the zfin.org database (http://zfin.org/ZDB-GENE-040420-1) [161] and in Ohlebusch et al. [7], and imply restricted expression patterns in distinct brain regions starting from segmentation stages. Moreover, *alpl* expression has been described within the mantle and interneuromast cells of the lateral line system of *Tg(alpl:mCherry)* transgenic lines [162] and in the retina of developing zebrafish embryos [163]. The neuronal function of Tnap in the central nervous system of zebrafish is so far unknown, but our inhibitor experiments indicate an influence on axonal growth within the midbrain [7].

### 6.2. Neurological Symptoms of HPP

Diverse neurological symptoms of HPP can occur at the onset of the disease, including depression, anxiety disorders, sleep disturbance, and epileptic seizures, and are indicating an influence of TNAP on the physiology of the nervous system [164]. Epileptic seizures are a symptom that is occurring in severely affected infants but not in adults [147,164,165]. Additionally, cystic encephalopathy and immense destruction of the cerebrum and the basal ganglia related to TNAP deficiency were described in a severe HPP case [147]. Issues like depression, anxiety disorders, and sleep disturbance are symptoms that are more relevant in adult patients [164]. Moreover, the occurrence of fatigue and headaches was recognized in more than 60% of the analyzed HPP patients, which is a higher prevalence than in the general U.S. population, which was used as a reference group in the study [164].

### 6.3. Localization of TNAP/Tnap in the Nervous System

Initially, the localization of TNAP/Tnap in brain regions of several vertebrate species has been investigated and compared to humans to conclude possible neurological functions of the enzyme in the nervous system and to determine similarities/differences between species. In most vertebrate species, the bone variant of TNAP is equivalent to the one that is expressed in brain tissues [166]. Interestingly, primates show a very distinct pattern of TNAP expression in their cerebral cortex, which is partially dependent on neuronal activity [167]. In the human neocortex, TNAP is expressed specifically in layer 4 of the primary visual and somatosensory cortices and layer 5 of the occipital, frontal and temporal lobe [168]. In mice, *Alpl* expression levels are higher during brain development at embryonic stages than later in the adult brain [154]. In the adult rodent brain, positive *Alpl* expression can be verified in many areas, i.e., in the cortex, the thalamus, the hypothalamus, the septum, and the olfactory bulb [155,169]. Intense TNAP signals can be attributed to blood vessels and spots of adult neurogenesis, like the subventricular zone (SVZ) [154,155,169].

We recently published a detailed analysis of *alpl* expression and Tnap activity in the zebrafish brain. In particular, expression of the tissue-nonspecific alkaline phosphatase is detected in distinct areas of the telencephalon and the mesencephalon of zebrafish [7]. Interestingly, *Alpl* expression in ventricular zones has previously also been described in mouse brains [155], which is consequently implicating that the enzyme may be involved in adult neurogenesis. Zebrafish display pronounced adult neurogenesis and regeneration potential and thereby may be utilizing Tnap function predominantly during adult stages, too [170,171,172]. The detailed function of Tnap in distinct zebrafish brain cells, either in neurons, synapses or in glia, is still elusive, but a large-scale proteomics experiment on zebrafish synapses identified TNAP/Tnap as an evolutionarily conserved protein within the postsynapse in vertebrates (https://www.genes2cognition.org/publications/zebrafish-prot/) [173].

### 6.4. TNAP/Tnap’s Influence on Pain, Inflammation, Purinergic Signaling, and Neurotransmitter Synthesis

To date, a couple of publications are dealing with the effects of TNAP on the development and the functionality of the nervous system, like synaptogenesis, myelination [146,174], neurotransmitter synthesis [175], and neurite outgrowth [176]. As explicitly reviewed in a publication by Street and Sowa, TNAP influences the regulation of pain sensation by metabolizing ATP, which exhibits pro- and adenosine anti-nociceptive effects due to its enzymatic activity as an ectonucleotidase [177]. Going along with this, HPP patients often suffer from chronic pain that is, among others, treated with nonsteroidal anti-inflammatory drugs (NSAIDs) [178], but can only partially be attributed to an increase of inflammatory processes in their body as pain from prevalent (pseudo)fractures is also decreasing the patients’ quality of life [179]. According to a study published by Seefried et al., pain is even the most prevalent symptom in the analyzed HPP patient cohort as more than 67% suffer from it [179].

**Figure 4:**
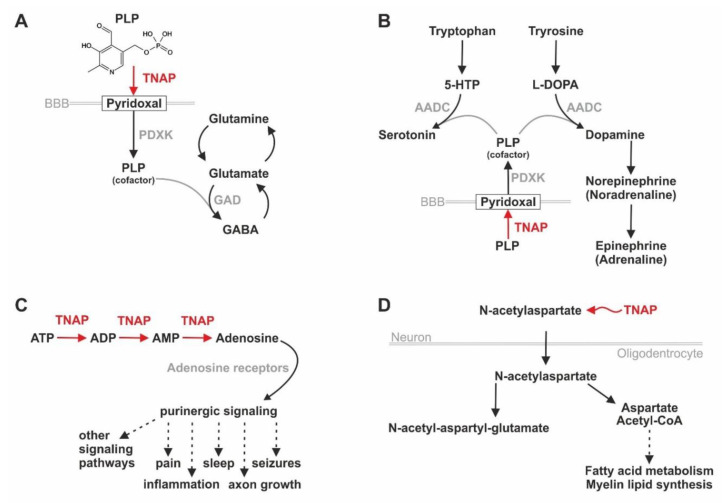
TNAP function is linked to PLP/cofactor, neurotrophic factor and myelin synthesis. (**A**) TNAP’s influence on the GABA synthesis. (**B**) TNAP’s role in the catecholamine and serotonin synthesis. (**C**) TNAP’s impact on purinergic signaling. (**D**) TNAP’s role in the myelin synthesis pathway. HTP: 5-hydroxytryptophan, AADC: aromatic L-amino acid decarboxylase, ATP/ADP/AMP: adenosine tri-/di-/monophosphate, BBB: blood–brain barrier, GABA: gamma-aminobutyric acid, GAD: glutamate decarboxylase, L-DOPA: levodopa, PDXK: pyridoxal kinase, PLP: pyridoxal-5′-phosphate, TNAP: tissue-nonspecific alkaline phosphatase.

For further insights into TNAP’s involvement in numerous pathways in the nervous system, like the purinergic signaling, which affects the functionality of diverse neuronal processes, we recommend reading the review by Negyessy et al. [180]. Figure 4 provides a summary of TNAP’s influence points on different molecular pathways in the nervous system. Here, Figure 4A,B are visualizing that TNAP activity is needed for de-phosphorylating PLP to PL, which is the transportable form of vitamin B6, in order to provide a sufficient amount of PLP in the brain, an essential cofactor for enzymes catalyzing the synthesis of diverse neurotransmitters [175]. Additionally, TNAP can degrade ATP in a stepwise manner to adenosine and, therefore, influence the availability of ligands for purinergic receptors, which is leading to effects on pain, sleep, inflammation, and other processes [148,177] (Figure 4C).

In order to analyze whether TNAP’s effects on the prevalence of cofactors (see Figure 4A,B) are indeed leading to differences in metabolites in the brain, Cruz et al. performed a comparison of *Alpl* k.o. (*Akp2^−/−^)* with heterozygous *Akp2^+/−^* mice and wildtype controls [174]. They detected significant differences in the measurable concentration of the two important neurotransmitters γ-aminobutyric acid (GABA) and adenosine as well as of N-acetylaspartate (NAA) and N-acetyl-aspartyl-glutamate (NAAG), which are both involved in myelin synthesis, also in rodents (see Figure 4D) [174]. Vitamin B6 deficiency may cause the early death of TNAP deficient mice due to seizures [68]. The molecular reasons behind this observation were described in two human neonates implying that TNAP dysfunction leads to a deficit of PLP in the brain because only the dephosphorylated form PL (transport form of vitamin B6) can cross the blood–brain barrier (BBB) and enter the brain prior to getting rephosphorylated there [175]. Consequently, neurotransmitter metabolism is disturbed due to the lack of the important enzymatic cofactor PLP in the brain as a secondary effect of TNAP dysfunction (see Figure 4A,B) [175]. Interestingly, the lack of PLP as a cofactor for the conversion of glutamate to GABA finally leads to a decreased prevalence of GABA in the brains of *Akp2^−/−^* mice, which is promoting epileptic seizures [174]. Additionally, purinergic signaling is also playing a prominent role in epileptic seizures, which may occur in severely affected HPP patients, via the ATP-binding receptor P_2_X_7_ [181]. As described previously, HPP-related neonatal seizures are considered to be vitamin B6-dependent due to the PLP lack causing a GABA deficit in the brain according to mouse experiments [68,174]. Interestingly, vitamin B6 blocks the activity of P_2_X_7_ receptors [181]. Due to TNAP being involved in the purinergic signaling by providing/removing ligands for purinergic receptors as well as in the vitamin B6 signaling by dephosphorylating PLP to PL, one could speculate on a role of TNAP as a connecting factor between several different signaling pathways in the nervous system, which was already stated by Negyessy et al. [180]. The authors performed a molecular network analysis using the network of signal transduction of a pyramidal neuron and information on TNAP gained from databases and literature research [180].

Of note, enduring stimulation of P_2_X_7_ receptors with a high concentration of ATP (e.g., due to CD39 dysfunction) leads to the desensitization of the signal transmission at the neuromuscular junction [152]. Furthermore, *Alpl* is expressed in neuronal cells of the dorsal horn in the spinal cord in their murine model and playing a role in pain reception via degradation of ATP to adenosine, which is, later on, inhibiting A1R receptors [149,177].

## 7. TNAP beyond Mineralization—The Role of TNAP for Sensory Perception

### 7.1. Visual Perception

Kantor et al. describe TNAP activity in the eye localized to vessels of the retina, the photoreceptor layer, and mostly in the outer and inner plexiform layers within many species [182]. TNAP/Tnap is present in the sublayers of the inner plexiform layer, especially in rats and zebrafish, respectively [182,183]. Nevertheless, to the best of our knowledge, no impairment concerning vision ability has been reported in the context of HPP patients so far, which may suggest the presence of a compensatory mechanism in the human eyes. Moreover, phenotypes concerning the vision and/or hearing abilities are also not prominent in the TNAP deficient mouse models (Mouse Genome Informatics database (MGI)) that have already been described in a previous chapter [70,85,146,181]. However, in the majority of the performed murine studies, the focus was probably put on bone, dental, and neurological phenotypes rather than sensory perception [70,73,85,86,110,111,146,184]. In contrast to humans and rodents, the zebrafish retina owns the capacity of life-long regeneration [185]. We detected *alpl* expression in the lens and the retina of zebrafish embryos in our whole-mount ISH study and received positive signals in the eyes of adult fish using qPCR and activity-dependent staining methods [7]. Overall, the eyes show the highest expression levels of *alpl* compared to all other investigated tissues of the adult zebrafish, suggesting an involvement in cell proliferation within the retina and potentially also in associated parts of the CNS. However, TNAP/Tnap inhibitor experiments in zebrafish embryos and literature research on other vertebrate species did not identify clear links between alkaline phosphatase expression and retinal development.

Observations in patients and k.o. animals imply that ATP released from Müller glia cells within the eye can be degraded to adenosine in the extracellular space even without TNAP function [186]. One possible candidate for providing such a compensatory mechanism, at least in our opinion, could be the ectonucleoside triphosphate diphosphohydrolase NTPDase1 (also called CD39) as this enzyme degrades ATP to AMP [186,187]. This may prevent extensive over-activation of the P_2_X_7_ receptor in neurons and photoreceptors, which would otherwise lead to subsequent cell death in the retina, as analyzed in the retina of mice and rats [187,188]. Additionally, P_2_X_7_ receptors are also present in human Müller glia, which are involved in regulating the activity of synapses via the release of ATP (e.g., due to stretching of the cell’s membrane) and adenosine [186,187]. Nevertheless, as NTPDases only degrade ATP to AMP, 5-ectonucleotidases like CD73 are additionally needed for the supply of adenosine, whereas TNAP generally degrades ATP to adenosine in three subsequent steps [150]. Interestingly, continuous treatment of HPP patients with asfotase alfa for at least four years may lead to ectopic calcifications of the conjunctiva in the eyes, which are, according to the authors, asymptomatic and not threatening the patients’ vision during treatment [189]. As we previously speculated on NTPDase1 providing a possible compensatory mechanism for TNAP dysfunction, it is interesting to mention that one of the k.o. mouse models (*Entpd1^tm1a(EUCOMM)Wtsi^*) developed an eye phenotype (however, only in male individuals) (http://www.informatics.jax.org/allele/ MGI:4433342). A detailed analysis of the mouse retina revealed that *NTPDase1*, besides its expression in blood vessels, is also detectable in the cellular processes of horizontal and ganglion cells [151]. Ricatti et al. suggest that the locally restricted expression of *NTPDases* enables a very specific control of ATP, ADP, and AMP in the extracellular space, which is, among others, indispensable for distinct communication processes between neuronal and glial cells [151]. Moreover, compensatory enzymes, like NTPDase 1 (ENTPD1/CD39), can be detected in photoreceptors and ganglion cells in zebrafish as well [151,190].

### 7.2. Sound Perception

The only sensory perception impairment that has already been described related to HPP is hearing disability. 33% of the HPP patients, which were analyzed in a retrospective chart review, suffered from hearing loss, which equals a prevalence ratio of 1.6 compared to the general U.S. population [164]. The reason for this may be aberrant concentrations of phosphate and calcium due to TNAP deficiency [164], which may cause mineralization defects of the auditory ossicles. A recent case report on an HPP patient suffering from perinatal subtype describes an improvement of his hearing ability under asfotase alfa treatment [191]. This supports the assumption that the lack of TNAP activity may indeed be causative for the hearing loss in this patient [191]. In line with these observations, the lateral line system in the zebrafish has been described as a second sensory organ expressing high levels of *alpl* by transgene expression (*Tg(alpl:mCherry)* ZFIN ID: ZDB-TGCONSTRCT-140,715–1). The *mCherry* expression driven by an *alpl* promoter fragment has been successfully used to investigate the function of specialized cells during the development of the lateral line organ, which corresponds to an ear-like structure in fish for sensing external sound stimuli and water movements, in zebrafish [192,193]. More precisely, in zebrafish, *alpl* expression has been exclusively described in mantle and interneuromast cells within the lateral line [162]. Mantle cell-enriched transcripts have been identified, and translational responses to hair cell ablation have been described in zebrafish [162,192]. Visualization of these regenerative processes by advanced cell-tracking and machine learning aided quantification in zebrafish larvae revealed how these processes are cellularly and temporally organized [193]. However, the biochemical function of Tnap, especially in the mantle and in supporting cells, has not been clarified yet.

### 7.3. Olfactory Perception

Tnap function has been associated with another sensory organ in zebrafish, the olfactory epithelium [194], which has not been reported in HPP patients. *alpl* expression localizes specifically to non-neuronal cells within the epithelium, needed for olfactory sensing, and has been linked to a novel adenosine receptor A2c in zebrafish [194]. The olfactory pit of zebrafish is thought to act as a sensitive sensor for ATP within the surrounding water. Within the nostril, Tnap converts extracellular ATP to adenosine and thereby activates downstream neurological circuits, which enable efficient olfactory sensing and foraging behavior of the zebrafish [194]. Furthermore, findings in *Alpl* k.o. mice suggest that the enzyme is important for NSC proliferation and differentiation of neuronal precursors that migrate towards the olfactory bulb and that deficits can (at least in vitro) be compensated by adding soluble enzyme [133,154,157,195].

## 8. Conclusions and Outlook

Taken together, the current level of knowledge implies that TNAP is involved in a wide number of molecular and biochemical processes and thereby acts as an important gatekeeper of physiological conditions in health. Under disease conditions, like the rare disease HPP, TNAP deficiency can lead to a prominent skeletal phenotype as well as a multisystemic disorder additionally affecting muscles, kidneys, lung (not discussed in this Review Article, for detailed clinical information see, e.g., [34,196]), teeth, and the nervous system. Most times, these far-reaching effects in HPP patients are caused by modulation of biological environments due to loss of TNAP function, like a lack of mineralization substrates in calcifying tissues like bone [1]. Additionally, a lack of biochemical substrates or essential enzymatic cofactors, like restricting PLP/vitamin B6 availability, are further contributing to the HPP phenotype [42].

Due to the high level of evolutionary conservation of the *ALPL* gene in different vertebrate species and due to the potential conservation of TNAP function [8], the opportunity to investigate its expression and its distinct functions in non-humans is apparent [129,166]. Eminently, solid validation of general molecular concepts due to interspecies comparison is possible by rebuilding HPP in different laboratory animal models, like rodents and zebrafish (see Table 1). The comparability of the experimental results gained from humans, rodents, and zebrafish, indicates that the established in vivo animal models are suitable for comprehensive research on different topics, e.g., bone development, cell signaling, and biochemical homeostasis. Table 1 summarizes information sources and key publications on *ALPL/Alpl/alpl* expression and TNAP/Tnap function in different vertebrate species.

Even though discussing the full range of the pathological potential of TNAP dysfunction would go beyond the scope of our review, which is mainly focusing on basic science and not on clinical results, we still want to accentuate that TNAP function could be correlated to a number of aging-associated diseases. These comprise, among others: Alzheimer’s disease and arteriosclerosis [201,202]. Future investigation of TNAP’s long-term effects in the course of aging societies worldwide and the linked health problems is imminent and should aim for a better understanding of sub-syndromic functional changes and potential medications. Future animal models will be established for further deciphering the molecular aspects of TNAP/Tnap function and will help to resolve these open questions experimentally.

## Figures and Tables

**Table 1:** Comparison of *ALPL/Alpl/alpl* expression and of TNAP/Tnap function in different species.

	Human	Mouse and Rat	Zebrafish and Other Vertebrates
*ALPL/Alpl/alpl* Expression			
**Expression database links**	The Human Protein Atlas (https://www.proteinatlas.org/ENSG00000162551-ALPL)	Mouse Genome Informatics (http://www.informatics.jax.org/marker/MGI:87983) Rat Genome Database (https://rgd.mcw.edu/rgdweb/report/gene/main.html?id=2100)	Zebrafish Information Network (https://zfin.org/ZDB-GENE-040420-1) *Xenopus*/Xenbase (http://www.xenbase.org/gene/expression.do?tabId=1&method=displayGenePageExpression&geneId=1010301&objId=1010301) Chicken/Geisha (http://geisha.arizona.edu/geisha/search.jsp?search=NCBI+ID&text=396317)
**Embryonic development**	Expression of *Alpl/alpl* has been intensively studied in mice and to a smaller extent in zebrafish, *Xenopus* and chicken embryos. It has been detected in developing brain regions (telencephalon and diencephalon), bone structures, genital organs, muscle/myotome, kidney, and heart.		
	N/A	[197]	[7]
**Bone and mineralizing structures**	Expression in skeletal bone and other mineralizing structures, e.g., teeth, has been reported for humans and rodents. In addition, expression in limbs/fins has been reported in mice and zebrafish.		
	[101]	[112]	Expression database
**Neurons and brain**	Expression in distinct brain regions is common to all vertebrates. Most prominently detected in the forebrain/telencephalon, epiphyses, and layers within the human neocortex.		
	[168]	[158]	[166,167]
**Eye**	Expression in the retina is reported for a wide number of vertebrates and is evolutionary highly conserved.		
	[183]	[183]	[183]
**Consequences of TNAP/Tnap Loss of Function**			
**Hypophosphatasia**	A large number of disease-causing mutations have been described and result in HPP in humans. Similar disease phenotypes have been reported in knockout and mutated mice, including decreased circulating TNAP levels in blood. Variable severity grades can be observed and result in different pathological disease classes in human patients and murine models.		
	[32,33]	[68,70,129]	N/A
**Defects in bone and mineralizing structures**	Changed levels of TNAP activity result in prominent skeletal and mineralizing defects in humans and mice, e.g., decreased bone density, lack of dentin mineralization, and increased bone resorption. Blocking of Tnap results in a delay of mineralization in zebrafish embryos.		
	[198,199]	[70,129]	[7]
**Defects in teeth**	Lack of mineralization results in abnormal tooth development, morphological deformations, and short tooth roots in humans and mice. Observed changes are *ALPL/Alpl* mutation-dependent.		
	[97,102,103]	[73,98]	N/A
**Defects in neurons and Brain**	Most prominently, neurological symptoms of HPP patients are seizures, anxiety disorders, and depression. In murine knockout models and heavily affected patients, seizures can be lethal. Although, neuronal tube defects, demonstrated by differences in spine nerve morphology and lumbar nerve roots development, can be abnormal in knockout mice.		
	[147,164]	[69,146,155]	N/A
**Craniosynostosis**	Fusion of sutures has been reported in human HPP patients and in knockout mice. However, the phenotype is mutation-dependent and highly variable.		
	[200]	[72]	N/A
**Vascular calcification**	HPP patients do not develop vascular calcification, although transgenic mouse models can show pathological arterial calcification.		
	N/A	[201]	N/A

N/A: not available.
